# Phase II study of liposomal doxorubicin and gemcitabine in the salvage treatment of ovarian cancer

**DOI:** 10.1038/sj.bjc.6601284

**Published:** 2003-09-30

**Authors:** G D'Agostino, G Ferrandina, M Ludovisi, A Testa, D Lorusso, N Gbaguidi, E Breda, S Mancuso, G Scambia

**Affiliations:** 1 Department of Gynecology Oncology, Catholic University of the Sacred Heart, Largo A. Gemelli, 8, Rome 00168, Italy; 2 Department of Medical Oncology, Ospedale Fatebenefratelli Isola Tiberina, Rome, Italy

**Keywords:** gemcitabine, liposomal doxorubicin, ovarian cancer

## Abstract

In total, 70 patients were enrolled into this phase II study, to evaluate the activity of the pegylated liposomal doxorubicin (PLD) and gemcitabine (GEM) combination in recurrent ovarian cancer patients. PLD, 30 mg m^−2^, was administered on day 1 by 60′ i.v. infusion, followed by GEM, 1000 mg m^−2^, given by 30′ i.v. on days 1 and 8; cycles were repeated every 21 days. In all, 67 patients are so far evaluable for response. Seven complete responses (10.4%, 95% CI: 3.1–17.7), 16 partial responses (23.9%, 95% CI: 13.7–34.1), 26 disease stabilisations (38.8%, 95% CI: 27.1–50.5) and 18 progressions (26.9%, 95% CI: 16.3–37.5) have been registered. Within the resistant population (*n*=36), the response rate was 25% (95% CI: 10.9–39.1). Within the group of platinum-sensitive patients (*n*=31), the response rate was 45.2% (95% CI: 27.7–62.7). A total of 443 courses are evaluable for toxicity. Grade 3–4 hematological toxicity was registered in 30 patients (42.8%), mainly represented by neutropenia (35.6%); palmar-plantar erythrodysesthesia affected 24 patients (34.2%), but it was of grade 3 in only seven of them (10%).

Over the last decade, the improvement of cytoreductive surgical efforts and the introduction of paclitaxel in the upfront platinum-based chemotherapy have increased the progression-free and overall survival of ovarian cancer patients (Berek *et al*, 1999). However, the recurrence of disease remains the main problem of ovarian cancer management, since most patients still die from the disease within 5 years of their initial diagnosis (Jemal *et al*, 2003).

With the exception of patients with long treatment-free intervals who can benefit from a platinum rechallenge and have a better prognosis (Markman *et al*, 1991; Ozols, 2000), for the vast majority of recurring ovarian cancer patients, palliation is the real goal of the second line therapy (Ozols, 2002a), in spite of the great amount of new drugs with significant activity which have been identified in recent years (Ozols, 1997, 2002b). In fact, the largest trials that have recently tested the most promising new drugs such as paclitaxel, topotecan, etoposide, liposomal doxorubicin and gemcitabine, in relapsed ovarian cancer, have demonstrated response rates ranging from 10 to 30% (Ten Bokkel Huinink *et al*, 1997; Rose *et al*, 1998; Gordon *et al*, 2001; Markman 2002). The use of drug combinations, which are considered a gold standard in the first-line approach (Berek *et al*, 1999; Ozols, 2000), is usually discouraged in the recurrent setting because of higher toxicity, and the lack of any evidence of benefit in terms of survival (Sabbatini *et al*, 1998; Colombo *et al*, 1999); nevertheless, the better toxicity profile expressed by some new categories of drugs allows the hypothesis that the rationalised choice of drugs with different mechanisms of action and toxicity patterns might increase the chances of response and favourably affect the clinical outcome. In this context, the combination of two of the above-mentioned drugs, namely GEM and PLD seemed particularly intriguing for several reasons: (i) both the drugs have shown activity in ovarian cancer (Hansen *et al*, 1999; Gordon *et al*, 2000, 2001; Markman, 2002); (ii) their different mechanisms of action are likely to hamper a cross resistance; (iii) the combination of GEM and doxorubicin has been reported to result in synergistic antiproliferative activity *in vitro* (Zoli *et al*, 1999; Chow *et al*, 2000); (iv) finally, the nonoverlapping toxicity profiles of GEM and PLD warrant the analysis of their combination in the clinical setting.

On the basis of the above considerations, we recently published the results of a phase I study aimed at determining the maximum tolerated doses (MTD) and toxicity of the GEM–PLD combination (D'Agostino *et al*, 2002a). The MTD was reached at the doses of PLD, 30 mg m^−2^, and GEM, 1000 mg m^−2^, the DLT being represented by febrile neutropenia and thrombocytopenia. In the small subset of patients enrolled in the phase I, a response rate of 21% was registered, which seemed to us quite encouraging considering that the majority of those patients were undertreated as often happens in phase I studies which do not allow an intraindividual dose escalation (Mathe and Reizenstein, 1986). These findings prompted us to accomplish this phase II study, in order to confirm the promising trend in a wider subset of recurrent ovarian cancer patients treated at the MTD, and to assess also the safety of the treatment in terms of haematological and nonhaematological toxicity.

## MATERIALS AND METHODS

### Eligibility

Patients with progressing/recurring epithelial ovarian cancer, previously treated with at least one platinum/paclitaxel chemotherapy regimen, and with radiological evidence of measurable (>2 cm) lesions were eligible for the study. Further entry criteria were: age over 18 years, Eastern Cooperative Oncology Group (ECOG) performance status ⩽2 (Oken *et al*, 1982), life expectancy >12 weeks, absolute neutrophil count (ANC) >1.5 × 10^9^ l^−1^; platelet count >150 × 10^9^ l^−1^; bilirubin and creatinine levels less than 1.5 times the upper limit of normal; normal cardiac function defined as LVEF ⩾50%. Patients were ineligible in the case of: previous or current malignancies at other sites with the exception of basal or squamous cell carcinoma of the skin and cone biopsed carcinoma *in situ* of the uterine cervix; Brenner's and borderline ovarian tumors; prior GEM or PLD chemotherapy or anthracycline therapy with a cumulative doxorubicin dose exceeding 300 mg m^−2^ or a cumulative epirubicin dose exceeding 540 mg m^−2^; significant heart disease including any history of ischaemic heart disease, any history of arrhythmia requiring treatment, or clinically significant valvular disease; other investigational cytotoxic drugs given within 30 days prior to entry into the study; symptomatic CNS metastases; uncontrolled severe infection and/or medical problems unrelated to malignancy which would limit full compliance with the study or expose the patient to extreme risk.

### Study design

This was a noncomparative phase II study of the combination of PLD and GEM. The approval of the local ethic committee was obtained prior to start of the trial. Before study entry, a written informed consent was requested from all patients. Within 14 days from the beginning of the study treatment, patients were submitted to a complete clinical evaluation (including CT-scan), laboratory tests, with complete blood cell count, serum chemistry, Ca 125 level and urinalysis, and echocardiography for the assessment of the baseline left-ventricle ejection fraction (LVEF).

PLD, 30 mg m^−2^, was administered on day 1 by 60′ i.v. infusion, followed by GEM, 1000 mg m^−2^, given by 30′ i.v. on days 1 and 8; cycles were repeated every 21 days. All patients received an antiemetic prophylaxis (metoclopramide) prior to the application of chemotherapy. Complete blood count and platelets were performed on a weekly basis; a routine 12-channel biochemistry was performed on days 1 and 14 of each cycle, unless differently indicated clinically. LVEF was evaluated every two cycles of chemotherapy by echocardiography. A multigated angiogram (MUGA) was planned if the echocardiography registered an LVEF decrease of >10%. Chemotherapy-induced toxicity was graded according to the National Cancer Institute common toxicity criteria (NCI-CTC, 1998). In the case of haemoglobin < 9 g dl^−1^, ANC < 1000 *μ* l^−1^ and/or PLT < 100 000 *μ *l^−1^, day-8 GEM administration was dropped or day-1 PLD/GEM treatment postponed by 1 week. In patients who had delayed treatment for more than 2 weeks and in the case of development of hypersensitivity reactions, treatment was discontinued. In the presence of grade 4 haematological toxicity, the doses of GEM and PLD were reduced by 20% in the next cycle. In the case of hand–foot syndrome (PPE), the dose of PLD was reduced by 20% in the next cycles. GSF and/or epoetin were administered in the cases of haematological toxicity according to the ASCO guidelines (Ozer *et al*, 2000; Rizzo *et al*, 2002).

Ca 125 levels were tested on day 1 of each cycle, clinical evaluation (including CT-scan) was planned every two cycles, and the clinical response assessed according to the RECIST criteria (Therasse *et al*, 2000).

## RESULTS

### Patient characteristics

From December 2000 to February 2003, 70 patients were enrolled into this phase II study. Patient characteristics are detailed in Table 1

**Table 1:** Patient characteristics

**Characteristic**	**No.**	**%**
Patients entered	70	100.0
Evaluable	67	95.7
Median age (range) (years)	59 (25–76)	
Median performance status (range)	1 (0–2)	
Platinum/paclitaxel resistant	38	54.3
Sensitive	32	45.7
		
*No. of prior chemotherapy regimens*		
1	34	48.6
2	26	37.1
3	6	8.6
>3	4	5.7

. The median age was 59 years (range 25–76).

In all, 38 patients were considered platinum resistant, that is, progressing during or within 6 months from the end of primary treatment with carboplatin and paclitaxel (or platinum rechallenge). A total of 32 patients were considered platinum sensitive since they had previously responded to platinum-based therapy and recurred more than 6 months after that treatment. Platinum rechallenge (10 patients, 31.2%) before the enrollment into this study was offered to those patients who relapsed more than 12 months after the completion of primary treatment. The median platinum-free interval was 3 months (mean 3 months, range 1–6) for the platinum-resistant patients, and 9 months (mean 13 months, range 7–39) for the platinum-sensitive patients.

### Response

In total, 67 patients are so far evaluable for response (95.7%, Table 2

**Table 2:** Clinical response according to platinum sensitivity

		**Complete**		**Partial**		**Stable disease**		**Progression**	
**Platinum sensitivity**	**Total**	**No.**	**%**	**No**	**%**	**No.**	**%**	**No.**	**%**
Resistant	36	1	2.8	8	22.2	13	36.1	14	38.9
Sensitive	31	6	19.4	8	25.8	13	41.9	4	12.9
Total evaluable pts	67	7	10.4	16	23.9	26	38.8	18	26.9

). Of them, two patients died due to the early progression of the disease after only one cycle, and one patient, previously rechallenged with carboplatin, experienced thrombocytopenia persisting for more than 2 weeks after the first cycle, and discontinued treatment.

In the overall series, seven complete responses (10.4%, 95% CI: 3.1–17.7) and 16 partial responses (23.9%, 95% CI: 13.7–34.1) have been registered, with an overall response rate of 34.3% (95% CI: 23.0–45.6). The median response duration was 22 weeks (range 4–85). Furthermore, 26 patients (38.8%, 95% CI: 27.1–50.5) experienced a stabilisation of the disease (median duration: 36 weeks, range 18–87). A total of 18 (26.9%, 95% CI: 16.3–47.5) progressed when on treatment. In the whole series, the median time to progression was 28 weeks (range 4–97). Within the resistant population, there were one complete and eight partial responses for an overall response rate of 25% (95% CI : 10.9–39.1). The median response duration was 18 weeks (range 4–50). Stabilisation of the disease was observed in 13 out of 36 patients (36.1%, 95% CI: 20.4–51.8) with a median duration of 36 weeks (range 18–87).

Within the group of platinum-sensitive patients, there were six complete and eight partial responses for an overall response rate of 45.2% (95% CI: 27.7–62.7). The median response duration was 28 weeks (range 4–85). Stabilisation of the disease was observed in 13 out of 31 patients (41.9%, 95% CI: 24.5–59.3) with a median duration of 35 weeks (range 18–71).

### Toxicity

A total of 443 courses are evaluable for toxicity, a median number of six cycles (range 1–13) having been administered per patient. The mean dose of PLD administered per cycle to each patient was 29 mg m^−2^ (median: 30 mg m^−2^, range 25–30 mg m^−2^), whereas the mean dose of GEM administered per cycle to each patient was 1932 mg m^−2^ (median: 2000 mg m^−2^, range 1520–2000 mg m^−2^).

A 20% dose reduction was required by 19 patients (27.1%), for a total of 78 cycles (17.6%); a 1-week-delay was necessary for five patients (7.1%) and a total of five cycles (1.1%). Seven patients (10%) required discontinuation of treatment because of haematological toxicity (*n*=3), palmar–plantar erythrodysesthesia (PPE) (*n*=1), gastrointestinal toxicity (*n*=1) and mucositis (*n*=2). Discontinuation due to PPE occurred after the fourth cycle, while in the other cases of nonhaematological toxicity, interruption occurred at the end of the 10th, second, and fifth cycles, respectively. In all, 15 patients (21.4%) (total number of cycles: 24, 5.4%) were unable to receive the day 8 GEM administration because of haematological toxicity.

Severe haematological toxicity was registered in 30 patients (42.8%, Table 3

**Table 3:** Haematological toxicity

	**Grade 3**				**Grade 4**			
**Toxicity**	**No. pts**	**(%)**	**No. cycles**	**(%)**	**No. pts**	**(%)**	**No. cycles**	**(%)**
Haemoglobin	3	(4.2)	3	(0.6)	2	(2.8)	2	(0.5)
Neutrophils	19	(27.1)	26	(5.8)	6	(8.5)	7	(1.5)
Platelets	5	(7.1)	6	(1.3)	1	(1.4)	1	(0.2)

); in particular, anaemia: G3, 4.2%; G4, 2.8%; neutropenia: G3, 27.1%; G4, 8.5%; thrombocytopenia: G3, 7.1%; G4, 1.4%. Blood transfusions were required in two patients (2.8%), and one of them was hospitalised because of severe fatigue. Eight patients (11.4%) were treated with epoetin. The granulocyte-colony stimulating factor (G-CSF), administered only in the case of G4 neutropenia with fever or persisting more than 5 days, was necessary for three patients (4.2%), and one of them was hospitalised. Thrombocytopenia was never complicated by bleeding episodes or platelet transfusion requirements.

The main nonhaematological toxicities are summarised in Table 4

**Table 4:** Nonhaematological toxicity

	**Grade 1**		**Grade 2**		**Grade 3**	
**Toxicity**	**No. pts**	**(%)**	**No. pts**	**(%)**	**No. pts**	**(%)**
PPE	6	8.5	11	15.7	7	10.0
Stomatitis	4	5.7	11	15.7	7	10.0
Paresthesia	3	4.2	2	2.8	1	1.4
Diarrhoea	5	7.1	3	4.2	1	1.4
Hepatic	2	2.8	3	4.2	0	0

: in no case did we observe grade 4 nonhaematologic toxicities or cardiac adverse events. PPE affected 24 patients (34.2%), but only in seven of them (10%) was it so heavy to compromise the activities of the day-living and only one (1.4%) required discontinuation of the therapy. Topic aloe-vera-based compounds were used to treat PPE, whereas oral dexamethasone (8 mg die^−1^) was administered only to the patients with grade 3 cutaneous toxicity. Mild stomatitis (G1–G2) was observed in 15 patients (21.4%). Paresthesia was registered in a total of six patients (8.5%). Nausea/vomiting was fully controlled by the metoclopramide premedication, whereas alopecia was not evaluated since the majority of the patients already presented with alopecia at the enrollment because of the previous treatments; anyway, it was not experienced *de novo* by any of the treated patients.

## DISCUSSION

We report herein the results of a phase II trial on the combination of GEM and PLD in recurrent ovarian cancer patients. In the whole series of patients, an overall response rate of 34.3% has been registered, with complete responders amounting to 10.4%. These findings are quite satisfying and confirm not only the encouraging results of the phase I but even the preliminary results of the phase II study recently presented at the 2002 ASCO meeting (D'Agostino *et al*, 2002b). Moreover, these results seem to represent the best response rates reported in the salvage treatment of ovarian cancer with regimens not including platinum and/or taxanes (Bajetta *et al*, 1996; Rose *et al*, 1998; Gordon *et al*, 2001).

Within the subgroup of resistant patients, the overall response rate of 25% is superior to the response rates of 18.3 and 12.3% obtained with PLD alone in phase II and III trials (Gordon *et al*, 2000, 2001), respectively, and is even better than the results obtained in several trials testing GEM in this clinical setting (Markman, 2002). Although it is recognised that the direct comparison of response rates across nonrandomized phase II studies is difficult, it is also worth noting that the percentage of stabilisation of the disease reported in the current study in the overall series (38.8%) and especially in the subgroup of resistant patients (36.1%) compare favourably to that reported by Gordon *et al* (2001) using PLD and by Shapiro *et al* (1996) using single-agent GEM.

The possibility to achieve a long-lasting disease stabilisation is an important end point for patients whose life expectancy is generally very poor, recent studies having demonstrated that the survival benefit following second line chemotherapy, if a complete remission is not obtained, is similar for partial responses and stable diseases, and that the distinction between both could be useless (Cesano *et al*, 1999).

Regarding the platinum-sensitive patients, we observed, as expected compared with the resistant patients, a higher overall response rate (45.2%), which is similar to the results obtained with platinum rechallenge or other cytotoxic agents (Markman *et al*, 1991; Bookman, 1999; Zanotti et al, 2000; Cannistra, 2002) and PLD, as previously reported (Gordon *et al*, 2001). This finding, together with the documentation of 41.9% of disease stabilisation in our series suggest that this drug combination might be of value in recurrent ovarian cancer patients considered sensitive to first-line platinum treatment. This issue seems quite intriguing in view of the potential clinical value of using nonplatinum drugs to prolong the platinum-free interval which is the most critical predictor of sensitivity to platinum rechallenge, although controversies still exist (Markman *et al*, 1991; Ozols, 1997; Bookman, 1999; Cannistra, 2002).

As far as the toxicity is concerned, the combination of GEM with PLD in a 21-day-based schedule led neither to the reduction of the mean (median) cumulative dose of PLD, which is quite comparable to that administered with the standard 50 mg m^−2^ 28-day cycle^−1^ (Gordon *et al*, 2000; 2001), nor did it imply a decrease in the dose intensity.

The proportion of patients experiencing G3/G4 neutropenia (35.7%) was considerable, and notably higher than that reported by using GEM or PLD alone; however, the rates of discontinuation, delay, interruption of treatment due to haematological toxicity were even lower than that reported in the above-mentioned studies (Shapiro *et al*, 1996; Gordon *et al*, 2001). Moreover, haematological toxicity was always easily managed and did not result in a larger use of haematopoietic growth factors, or blood transfusions, when compared with the regimens utilising PLD (50 mg m^−2^) in a 28-day based schedule (Gordon *et al*, 2001).

Regarding nonhaematological toxicity, stomatitis and PPE were, as expected, the most frequently observed adverse events. In particular, PPE, which is the primary dose-limiting side effect of PLD, occurred in a lower percentage of cases with respect to what was reported by the main trials testing PLD (Gordon *et al*, 2000, 2001; Safra *et al*, 2001), requiring the discontinuation of treatment in only 2.8% of cases. G3-G4 PPE, in fact, hit only 10% of patients in our series, compared with 18, 17.3 and 23% registered in the previously reported studies (Gordon *et al*, 2000, 2001; Safra *et al*, 2001), respectively.

A formal health-related quality of life research was not performed in this trial; we do believe that a disability paradox (Albrecht and Devlieger, 1999) may often induce the patients enrolled in a phase II study to accommodate to their illness, changing internal standards and values, and eventually overestimating the meaning of their self-evaluation of quality of life (Sprangers and Schwartz, 1999); anyway, the fact that a total of 443 courses have been administered in an outpatient setting with full compliance by the patients, who received a median number of six cycles each, witnesses the safety of the treatment.

In conclusion, the combination of gemcitabine and PLD has proved to be a valid approach in recurrent ovarian cancer patients. In particular, in the subgroup of resistant patients the overall response rate and, above all, the proportion of disease stabilisation were encouraging. Furthermore, the results reported in this study relaunch the option that a polychemotherapy can offer better chances of response to fit patients even in the salvage setting. In this context, in fact, some reports of high response rates for drugs used in combination (Dizon *et al*, 2001) and the recent availability of less-toxic drugs and better haematological supports make the use of combination chemotherapy in recurrent ovarian cancer, a re-emerging challenging issue.

Based on *in vitro* data suggesting a potential synergistic interaction between the two drugs (Zoli *et al*, 1999; Chow *et al*, 2000), specific tests in preclinical models are ongoing in our laboratories in order to analyse the antitumour activity of the GEM/PLD combination and possibly clarify the underlying mechanisms of drug interaction.
